# Interaction of silver nanoparticles with catechol *O-*methyltransferase: Spectroscopic and simulation analyses

**DOI:** 10.1016/j.bbrep.2021.101013

**Published:** 2021-05-11

**Authors:** Aminu Usman, Kevin Lobb, Brett I. Pletschke, Christopher G. Whiteley, Brendan S. Wilhelmi

**Affiliations:** a Department of Biochemistry and Microbiology, Rhodes University, P.O. Box 94, Makhanda, (Grahamstown), 6140, South Africa; b Department of Chemistry, Rhodes University, P.O. Box 94, Makhanda, (Grahamstown), 6140, South Africa

**Keywords:** Catechol *O-*Methyltransferase, Silver nanoparticles, Spectroscopic and *in silico* analysis

## Abstract

Catechol O-methyltransferase, an enzyme involved in the metabolism of catechol containing compounds, catalyzes the transfer of a methyl group between S-adenosylmethionine and the hydroxyl groups of the catechol. Furthermore it is considered a potential drug target for Parkinson’s disease as it metabolizes the drug levodopa. Consequently inhibitors of the enzyme would increase levels of levodopa. In this study, absorption, fluorescence and infrared spectroscopy as well as computational simulation studies investigated human soluble catechol O-methyltransferase interaction with silver nanoparticles. The nanoparticles form a corona with the enzyme and quenches the fluorescence of Trp143. This amino acid maintains the correct structural orientation for the catechol ring during catalysis through a static mechanism supported by a non-fluorescent fluorophore–nanoparticle complex. The enzyme has one binding site for AgNPs in a thermodynamically spontaneous binding driven by electrostatic interactions as confirmed by negative ΔG and ΔH and positive ΔS values. Fourier transform infrared spectroscopy within the amide I region of the enzyme indicated that the interaction causes relaxation of its β−structures, while simulation studies indicated the involvement of six polar amino acids. These findings suggest AgNPs influence the catalytic activity of catechol O-methyltransferase, and therefore have potential in controlling the activity of the enzyme.

## Highlights

• A recombinant soluble human catechol O-methyltransferase was inhibited by silver nanoparticles.
• Inhibition by AgNPs was concentration and size dependent.
• The binding mechanism was through spontaneous static quenching, driven by positive Δ*S*, and negative Δ*H* and Δ*G.*
• Stern-Volmer analysis suggested binding of AgNPs with Trp143.
• *In silico* indicate relaxation of β-sheets and the interaction of AgNPs with 6 amino acids in the enzyme’s helical structures.

## 1 Introduction

The physiological levels of catechol-containing compounds (CCC) are impaired in several disease conditions, including Parkinson’s disease (PD), breast cancer and several neuropsychological disorders [[1], [2], [3]]. Catechol *O*-methyltransferase (COMT) [EC 2.1.1.6] is a magnesium ion-dependent enzyme that catalyzes the transfer of a methyl group from S-adenosyl-*L*-methionine (SAM) to the hydroxyl moieties of a catechol substrate (Supplementary Fig. S1) [4]. It plays a vital role in the metabolism of both endogenous and exogenous bioactive and bio-toxic CCC such as catechol-neurotransmitters, catechol-estrogens and catechol-drugs [5].

Of importance among the catechol-drugs is levodopa, a dopamine precursor which not only crosses the blood brain barrier (BBB), but remains the most relevant pharmacological medication for PD [6]. Levodopa therapy is associated with dose-related nausea as a result of its peripheral conversion to dopamine by the aromatic amino acid decarboxylase (AADC) [7]. The metabolism of levodopa in the periphery also causes fluctuations, including the lowering of its effect and inconsistent symptomatic relief in addition to the development of dyskinesia, both increasing with the advancement of the disease. Co-administration of AADC-inhibitors is devised to block the peripheral metabolism by AADC and subsequently increase delivery of levodopa to the brain [8,9]. *O*-methylation to 3 *O-*methyldopa (3-OMD) by the soluble (peripheral) COMT becomes the major degradation pathway for levodopa in the periphery after the blockage of the decarboxylation by AADC-inhibition [7,10]. In addition, 3-OMD competes with levodopa for active transport across the BBB and thus decreases brain uptake of the drug [11], further emphasizing the significance of COMT as a target for therapeutic strategies.

The soluble COMT isoform occurs at high levels in the liver, kidneys and the gastrointestinal tract, in contrast to membrane-bound COMT, which is predominant in the brain [12]. Inhibition of peripheral COMT increases the levodopa available to cross the BBB. The brain penetrating tolcapone and peripherally acting entacapone are two major second generation COMT nitrocatechol-containing competitive inhibitors used as adjuncts to levodopa therapy in the management of PD and other neuropsychological disorders. A more recently approved third generation nitrocatechol peripheral acting COMT inhibitor, opicapone, has a longer half-life and the advantage of a low, single daily dose [13]. These drugs decrease the fluctuations in plasma levodopa levels and lower the daily levodopa requirements [14,15]. However, each of these drugs have limited use with their associated problems in pharmacokinetics, clinical efficacy or in toxicity. Therefore research for novel inhibitors of COMT is currently ongoing, with screening of natural compounds such as catechin and alizarin showing inhibition [16], and cerium oxide nanoparticles showing potential *in silico* [17].

Silver nanoparticles (AgNPs) possess large surface area to volume ratios, which accord them increased reactivity with their contiguous milieu, including bio-molecules [18]. Interaction of NPs with proteins is the basis of the NP bio-reactivity that culminates in the formation of a NP-protein corona [19]. The NP surface induces conformational changes on the adsorbed protein structure, which may subsequently affect its biological function. Although difficult to show directly, the induced conformational changes have important consequences, since the partial unfolding of protein domains expose hitherto hidden amino acid residues [19,20]. For example, lysozyme adsorbed onto the surface of NPs is reported to lose 10% of its secondary structure and to show a marked decrease in enzymatic activity [21].

Two forms of COMT have been identified in mammals, namely a membrane-bound (MB-COMT) and a cytoplasmic soluble (SCOMT) enzyme [22]. The human MB-COMT has an additional N-terminal extension of fifty hydrophobic amino acid residues relative to SCOMT [[23], [24], [25]]. The extension contains a signal-anchor region, facilitating the anchoring of the MB-COMT protein onto cellular membranes [23,24]. In our study, the effect of AgNPs on a recombinant human SCOMT was assessed. The mechanism of the AgNP-SCOMT interaction was evaluated using spectroscopic approaches and a molecular docking study.

## 2 Materials and methods

### 2.1 Materials

Except where otherwise stated, solvents and reagents used in this study were of analytical grade and were used as procured from the commercial suppliers without further purification. Milli-Q water dispensed by a Milli-Q Elix system (Merck) was used in all the experiments.

### 2.2 Methods

#### 2.2.1 Expression and purification of SCOMT

The amino acid sequence of SCOMT was obtained from the National Centre for Biotechnology Information (accession code: NP_009294.1). The sequence was translated to its corresponding nucleotide sequence and optimized for *E. coli* expression [26]. The optimized gene was synthesized by GenScript (GenScript USA Inc.) and received in a lyophilized pET-22b(+) vector containing a C-terminal poly-histidine tag. The pET-22b(+) plasmid DNA harboring the SCOMT gene was sequenced to ascertain the correct gene insertion. A method previously described was adopted, with slight modification, for the expression of human SCOMT [26]. *E. coli* BL21 (DE3) cells were transformed with the pET-22b(+) plasmid harbouring the SCOMT gene and grown at 37 °C with shaking. When the cell cultures reached an absorbance of 0.7 at 600 nm, SCOMT expression was induced with 700 μM isopropyl β-d-1-thiogalactopyranoside and grown for an additional 4 h. Cultures were harvested by three rounds of centrifugation (6,000 × *ɡ*; 15 min) and washing (100 mM Tris-HCl buffer, 5 mM MgCl_2_, pH 7.5). The resultant pellet was resuspended (5% w/v) in the enzyme’s storage buffer (100 mM Tris-HCl buffer, 5 mM MgCl_2_, 5 mM ß–mercaptoethanol, 0.1 mM dithiothreitol pH 7.5). The cell suspension was lysed with 1 mg/ml lysozyme incubated at 37 °C for 1 h. The lysed culture was stored at −80 °C overnight to aid cell disruption. The cell extract was thawed on ice and centrifuged (15,000 × *ɡ*; 30 min) to remove cellular debris. To separate the soluble fractions, the supernatant was subjected to ultra-centrifugation (100,000 × *ɡ*; 60 min) and the resultant supernatant filtered (0.22 μm pore size, Millipore) and applied to a 5 ml HisTrap FF nickel affinity column (GE Healthcare) connected to an ÄKTA FPLC. Non-specifically bound proteins were eluted with 50 ml of wash buffer (100 mM Tris-HCl, 200 mM NaCl, 5 mM MgCl_2_, 5 mM β–mercaptoethanol, 20 mM imidazole, pH 7.5). His-tagged bound SCOMT was eluted in 5 ml fractions, using a 100 ml elution volume with a linear gradient of imidazole (0 – 400 mM) concentration. Fractions with higher SCOMT activity were pooled and concentrated to 2.5 ml and applied to a Sephadex G-25 column (GE Healthcare) equilibrated with 25 ml storage buffer (100 mM Tris-HCl buffer, 5 mM MgCl_2_, 5 mM β–mercaptoethanol, 0.1 mM dithiothreitol, pH 7.5) and eluted with the storage buffer in 10 × 1.0 ml fractions.

#### 2.2.2 Protein concentration

Protein concentration was routinely assayed by the Bradford assay [27], in triplicate with a 96-well microplate, using bovine serum albumin as standard. Protein (5 μl) was added to a well, followed by Bradford reagent (245 μl). The mixture was incubated at 22 °C for 10 min and absorbance of the solution measured at 595 nm.

#### 2.2.3 SDS-PAGE and Western blot

The presence and purity of SCOMT was assessed during the expression and purification processes by SDS-PAGE [28]. Samples (20 μl) and a standard protein molecular weight marker were electrophoresed on 12% SDS-PAGE at 120 V for 90 min. The gels were stained and destained using a modified Fairbanks method [29]. Separate gels (before staining) were transferred onto nitrocellulose membranes and the SCOMT was confirmed by Western blot, using the procedure of [30]. The molecular weight of the purified SCOMT was determined using a standard curve of log molecular weight versus distance migrated [31].

#### 2.2.4 SCOMT activity assay

Enzymatic activity of SCOMT was assayed based on a published protocol [32] with slight modification. The final reaction concentrations of the SCOMT preparation, the substrate esculetin, and the co-factor SAM in the reaction mixture were respectively, 5 μg/ml, 4 μM and 60 μM in a 200 μl reaction volume. Four-fold concentrations each of the final assay concentration of the enzyme preparation, esculetin, AgNPs and SAM were prepared in the enzyme activity buffer (100 mM phosphate, 5 mM MgCl_2_, 20 mM l-cysteine, pH 7.4). The substrate, esculetin, was dissolved in dimethyl sulfoxide (DMSO) and then diluted with the reaction buffer to a final DMSO concentration of 2% (v/v) (8% in four-fold concentration). Fifty microliters each of the four-fold reaction concentrations of enzyme preparation and esculetin were added to 50 μl of AgNPs and incubated at 30 °C for 60 min, before addition of 50 μl of the four-fold concentration of SAM to initiate the catalytic reaction. A control involved addition of 50 μl of the reaction buffer in lieu of AgNPs. A change in fluorescence (*λ*_ex_ 355 nm and *λ*_em_ 460 nm), caused by the enzymatic methylation of esculetin to scopoletin, was used as the index of SCOMT activity and was followed for 20 min with a Synergy Gen5 Multi-Mode Reader. The change in fluorescence of assay mixtures without esculetin, SAM and the enzyme preparation were included as controls.

To determine the pH optimum, the SCOMT activity was assayed in various buffer salts (sodium acetate (pH 4.5–5.5, 100 mM); Hepes (pH 6–7, 100 mM); phosphate (pH 7.4–8.5, 100 mM)). The temperature optimum of the purified enzyme was determined in the enzyme activity buffer over a range of 20 to 65 °C. The temperature stability of SCOMT was determined at the optimum temperature and pH and the relative SCOMT activity was calculated using the highest activity at a given time as the 100% activity and aliquots were removed at 15 min intervals and analyzed for enzyme activity for a maximum period of 2 h.

#### 2.2.5 Synthesis and characterization of AgNPs

AgNPs were synthesized using a microwave-assisted method [28]. A mixture of an ethanolic solution of polyvinylpyrrolidone PVP (1% w/v) and AgNO_3_ (0.002 M) was microwaved (LG; 720 W; 2, 450 MHz) for 7 s. The initially colourless solution turned to pale yellow, indicating the formation of AgNPs [26]. Plasmonic absorption of AgNPs was recorded using a BioTek Synergy MX (BioTek®) microplate reader. The wave scan was performed in triplicate between 300 and 700 nm using a 96 well micro plate. Wave scans of a PVP solution corresponding to its concentrations in Ag NPs were recorded as controls. A transmission electron microscope (TEM) (Zeiss Libra 120 keV coupled to an Olympus Soft Imaging Solutions digital camera) was used to measure the morphology and size of the particles. A drop of the AgNP solution was placed on a carbon-coated copper grid (Agar Scientific) and allowed to settle for 30 s. Excess was removed using blotting paper and the grid was air-dried for 24 h prior to TEM viewing. Size distributions of the NP were determined by counting the NPs in the TEM images using the ImageJ version1.42 software (Rasband, W. S; National Institutes of Health, Bethesda, Maryland, USA).

#### 2.2.6 Absorption spectroscopy

SCOMT at 0, 5, 10, 20, 40 and 80 μg/ml concentration was incubated (30 °C for 1 h in the dark) with AgNPs (250 μM) in a final volume of 1 ml in 1.5 ml reaction tubes. The plasmonic absorption spectra (300 to 800 nm) for AgNPs, as well as that for 80 μg/ml SCOMT, were recorded using a 96-well BioTek Synergy MX (BioTek®) microplate reader.

#### 2.2.7 Fluorescence spectroscopy

Emission spectra of SCOMT in the presence and absence of the NPs were measured using a PowerWave microplate spectrofluorimeter (BioTek Synergy MX, BioTek®) (equipped with Gen5 software) operated at 2.5 nm bandwidth. The excitation wavelength was 295 nm and the emission was recorded between 350 and 750 nm, in a buffer containing 100 mM Tris, 5 mM MgCl_2_, 200 mM NaCl, at pH 7.5. Five micrograms of SCOMT was mixed with 0, 10, 20, 30, 40, 50 and 60 μM of Ag NPs, in a final reaction volume of 200 μl; and incubated for 1 h with tin foil protection from ambient light. The incubations were performed at four temperatures: 25 °C (298 K) 30 °C (303 K), 35 °C (308K) and 40 °C (313K) before recording the fluorescence in a clear-bottomed black 96-well plate (Greiner Bio-one, Frickenhausen, Germany).

#### 2.2.8 Fourier transform infrared (FTIR) spectroscopy

FTIR was employed to study the interaction between Ag NPs with SCOMT. The reaction mixture contained 0.5 mg SCOMT in 60 μM AgNPs, in a final volume of 1 ml. These solutions (and SCOMT without AgNPs) were covered with tin foil, incubated (30^o^C, 1 h) then frozen at −80^o^C. After freezing, the samples were lyophilized using a Labconco FreeZone 6Plus freeze dryer (Vacutec, Johannesburg, South Africa) at 0.01 mBar, at −70 °C. The spectra of the lyophilized samples were obtained using a FTIR/ATR spectrometer Spectrum100 (PerkinElmer, Shelton, CT) in the frequency range of 4 000 to 500 cm^−1^ (2 cm^−1^ resolution) with air as background. Spectra were displayed in the transmittance mode and baseline corrected using PerkinElmer Spectrum software (Version 6.0.2.0025, PerkinElmer, Shelton, CT–USA).

#### 2.2.9 Computational simulation: molecular docking

The structure of SCOMT at 2.8 Å resolution bound to SAM and 3,5-dinitrocatechol (DNC) (PDB code: 3A7E) was used for binding studies. The silver nanoparticle was constructed within Discovery Studio Visualizer 4.0 (DSV4.0) from the crystal structure of silver, obtained from the Crystallography Open Database (COD) [33] deposited by Novgorodovo et al., (1979) [34] (COD ID No. 1509145). The unit cell was replicated in all dimensions, followed by exclusion of atoms to generate a sphere of atoms (radius = 30.0 Å, volume = 2 827.4 Å^3^). From this a sphere cap containing 277 silver atoms was used. Parameters for silver (R_ii_ = 3.15, ε_ii_ = 0.036) were included in an AD4 parameter file, used forAutoGrid4 calculations. The preparation of the pdbqt format of the AgNP sphere cap was through the use of a custom script, and not with prepare_ligand4.py, due to the size and rigidity of the system. Crystallographic water molecules and the co-crystallized ligands (SAM and DNC) were removed from the 3A7E crystal structure using DSV4.0, and this structure was prepared for docking using MGLTools 1.5.4, ensuring polar hydrogen atoms were present, and all other hydrogen atoms were merged, and that Marsilli-Gasteiger charges and Autodock4 atoms types were assigned for all atoms [35]. The grid box, with dimensions 49.026 Å, 51.0687 Å and 51.0687 Å, was centered in the SAM pocket of 3A7E and Autogrid4 was used to generate the appropriate grid maps. Docking was then performed using ten Lamarckian Generic Algorithm (LGA) runs, each with a population size of 100. The lowest energy bound AgNP was extracted and overlaid with 3A7E within DSV4.0 to evaluate and record the docking mode.

#### 2.2.10 Statistical analyses

All analyses were carried out in triplicate and values were reported as the means ± standard deviations. Where necessary, results were analyzed using GraphPad Prism software, version 5 and Microsoft Excel 2010.

## 3 Results and discussion

### 3.1 Expression and purification of SCOMT

Following the confirmation of the SCOMT gene insert by sequencing (Supplementary Fig. S2), the gene was expressed in *E. coli* BL21 DE3 harboring the vector pET-22b(+) + SCOMT. Approximately 11.51 g of cells were harvested from 1.8 L of the culture media, lysed and purified on a Ni-nitriloacetic acid affinity column. SCOMT was purified further (to remove the imidazole) on a Sephadex G-25 column (GE Healthcare), yielding a near homogeneous protein at a final purification fold and yield of 5.62 and 22.6%, respectively (Table 1). The specific activity of the recombinant SCOMT was 3.85 U mg^−1^ and the optimum pH and temperature values for the recombinant SCOMT were 7.0 and 30 °C, respectively.

**Table 1:** Purification of recombinant SCOMT.

Fraction	Volume (ml)	Protein concentration (mg/ml)	Total protein (mg)	Total activity (U)	Specific activity (U/mg)	Purification fold	% Yield
**Crude**	162	3.87	626.94	429.1	0.68	1	100
**Soluble fraction**	118	2.14	252.52	398.23	1.58	2.3	92.81
**Ni-affinity chromatography**	40	0.71	28.40	103.63	3.65	5.33	24.15
**Sephadex G25**	7.2	3.5	25.20	96.96	3.85	5.62	22.6

**Fig. 1:**
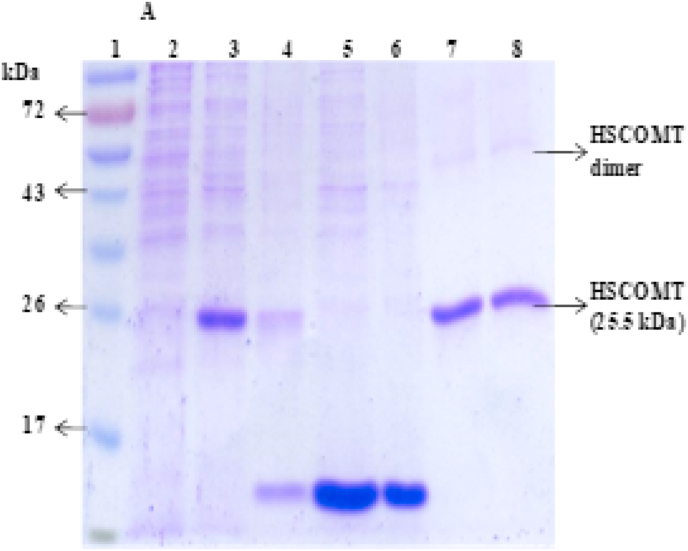
A SDS-PAGE gel of samples collected after each purification stage showing the protein ladder (1), grown *E. coli* cells harbouring empty (no SCOMT gene) plasmid (2), crude lysate sample (3), cleared lysate sample (4), IMAC flow-through sample (5), IMAC Wash sample (6), IMAC eluate sample (7) and size exclusion elute sample (8).

Prominent bands corresponding to the size of monomer (~25.5 kDa) of SCOMT appeared in both SDS-PAGE (Fig. 1) and Western blot images (Supplementary Fig. S3). Other bands corresponding to the sizes of the dimer and a larger oligomer (on Western blot gel image) were also observed (Supplementary Fig. S3). Oligomer formation by HSCOMT has been reported previously [36,37]. Cotton et al. (2004) reported that at high concentrations (≥1.2 mg/mL), SCOMT occurred as a mixture of the monomer and catalytically functional homo-dimer [36], and a larger oligomer [37], even under reduced SDS-PAGE conditions. Cotton and co-workers (2004) reported COMT methylation of catechols in both monomer and dimer forms of the enzyme, with the dimer possibly formed through intermolecular disulfide bonds between cysteines 188 and 191.

### 3.2 Characterization of AgNPs

A colourless mixture of ethanolic PVP (1% w/v) and AgNO_3_ (0.002 M) turned to pale yellow after seven (7) sec of microwaving, indicating the formation of AgNPs [38]. The exhibition of maximum absorption at 405 nm indicated the plasmon absorption of spherical AgNPs of ~10 nm diameter [39]. The shape, size and distribution of the synthesized silver NPs were visualized by TEM, which confirmed them to be spherical in shape with diameter sizes of 4 to 10 nm and stable in the dark at 23^o^C for up to 14 days (Supplementary Fig. S4).

### 3.3 Interaction of SCOMT with AgNPs: effect on enzymatic activity

**Fig. 2:**
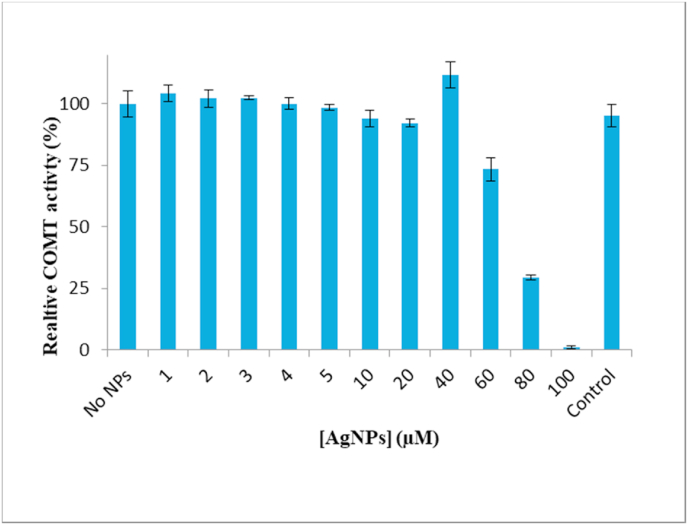
Relative activity of SCOMT with esculetin as the substrate and in the presence of varying amounts of AgNPs. Control is the SCOMT activity in the assay performed in the presence of equivalent concentration of ethanolic PVP and silver nitrate solutions used in the preparation of 100 μM AgNPs; while “0” represents the SCOMT activity without AgNPs (0.594 μmol ml^−1^ min^−1^) and was used as 100% reference standard for calculating the relative % activity. Values represent the mean (n = 3, ±SD).

The interaction of AgNPs with human SCOMT was determined (Fig. 2) by including the particles at a range of concentration of 0 to 100 μM in the enzyme activity assay with esculetin (4 μM) as a catechol substrate and SAM (60 μM) as a methyl donor. AgNPs up to 40 μM had no significant effect on SCOMT activity. However, SCOMT enzymatic activity decreased to 73%, 29% and 1% (relative activity) in the presence of 60 μM, 80 μM and 100 μM AgNPs, respectively. The SCOMT assay containing equivalent concentrations of ethanolic PVP and silver nitrate solutions used in the preparation of 100 μM AgNPs produced 95% catalytic activity, signifying that these solutions had limited effect on the SCOMT enzymatic activity at these concentrations. This is an indication that the decrease in SCOMT activity was caused by the presence of the AgNPs, although high ethanol concentrations (50 to 1,000 mM) have been reported to increase the enzymatic activity of COMT [40].

### 3.4 Interaction of SCOMT with AgNPs: spectroscopic analyses

#### 3.4.1 Absorbance spectroscopy

**Fig. 3:**
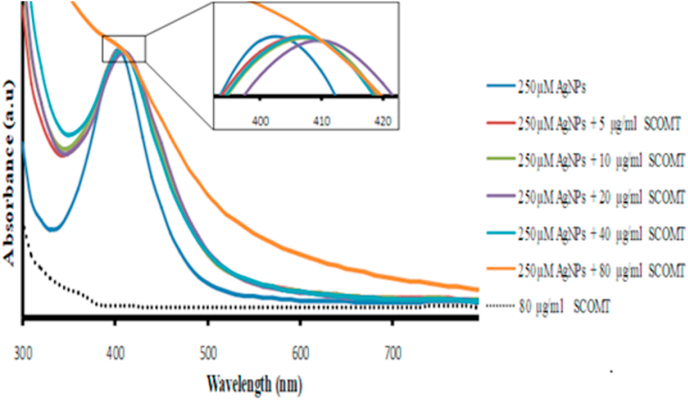
Normalized absorption spectra of 250 μM AgNPs from 300 to 800 nm, incubated in the dark for 1 h at 30^o^C, in the absence and presence of increasing concentrations of SCOMT. Inset is the enlarged plasmon peaks, emphasizing the broadening and bathochromic red shift of the peaks. The increase in SCOMT concentration correlates with red shift and increase in the peak broadening, indicating formation of AgNP-SCOMT corona. The discontinuous curve is the absorption spectrum of 80 μg/ml SCOMT at 300 to 800 nm. (For interpretation of the references to colour in this figure legend, the reader is referred to the Web version of this article.)

The plasmon band of AgNPs is sensitive to the interaction with protein and this criterion has been employed to monitor the interaction of NPs with proteins [41,42]. The plasmonic peak of AgNPs exhibited a SCOMT concentration-dependent red shift and broadening (Fig. 3), which indicates a direct conjugation of SCOMT to the particle surfaces. The shift and broadening of the plasmonic bands of the AgNP–SCOMT conjugates depend on the particles’ size and the concentration of the adsorbed protein [43] and reflects the formation of AgNP-SCOMT corona [41]. Incubation of 250 μM AgNPs with 80 μg/ml SCOMT decreased the plasmonic peak of the NPs. This can be attributed to the saturation of the protein binding positions, since PVP-coated NPs adsorb limited amounts of protein on their surfaces [44].

#### 3.4.2 Fluorescence spectroscopy

##### 3.4.2.1 Binding site of AgNPs on SCOMT

The binding parameters of AgNP for SCOMT can be analyzed from equation (1).

(1) Log[(Fo – F)/F] = logKa + nlog[Q]

where *Fo* and *F* are, respectively, the fluorescent intensities of SCOMT in the absence and presence of AgNPs; [Q] is the concentration of AgNPs; *n* is the number of binding sites (on the SCOMT) available for AgNPs, and *Ka* is the association constant (with its reciprocal as the dissociation constant (Kd)).

**Fig. 4:**
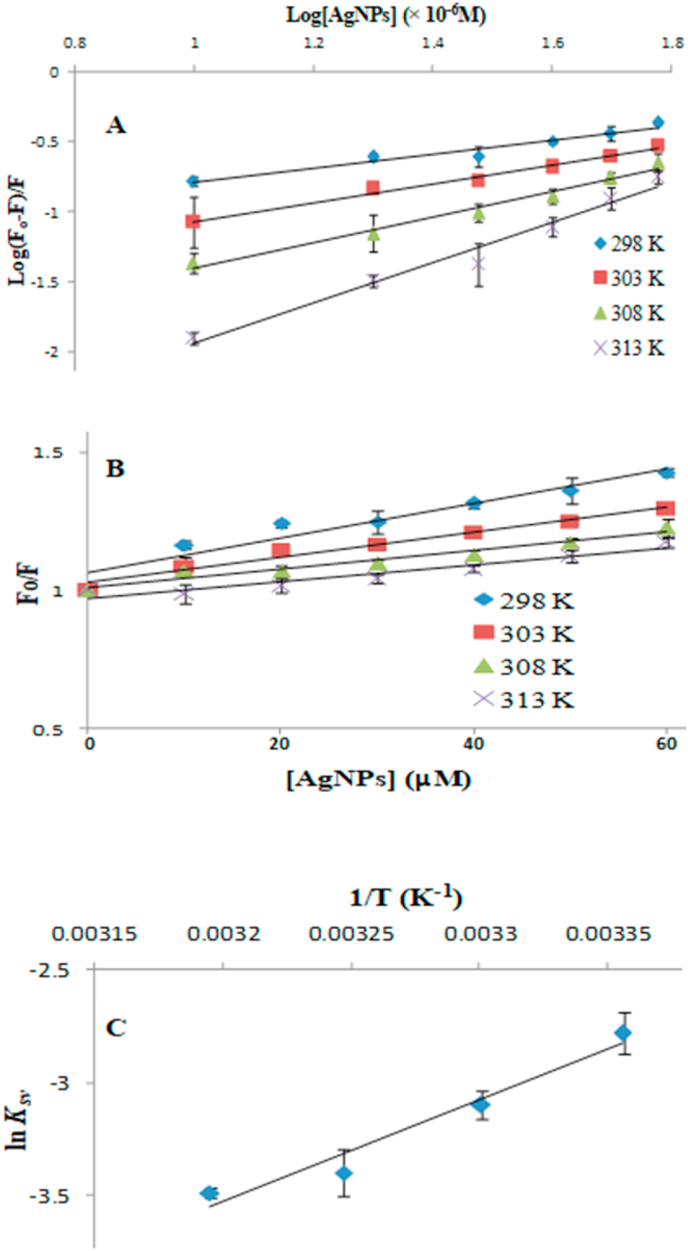
(A) Hill plot of log (*F*_*o*_*-F*)/*F* versus log [AgNP], (B) Stern–Volmer plot and (C) Van’t Hoff plot fluorescence (quenching) study of 5 μg SCOMT (5 μl) in 100 mM Tris, 5 mM MgCl_2_, 200 mM NaCl, pH 7.5 buffer, treated with AgNP [0 – 60 μM] in a final volume of 200 μl at temperatures 298, 303, 308 and 313 K. The *λ*_ex_ was 295 nm and the *λ*_em_ was 350 to 750 nm.

*Ka* is estimated from the intercept and *n* from the slope of the linear regression plot (Fig. 4A). There was only one available binding site for AgNPs on SCOMT. The values for the binding constants (*Kd*) of AgNPs-SCOMT binding increase with increase in temperature (298 to 313 K) indicating a strong affinity between AgNPs and SCOMT [45].

##### 3.4.2.2 Analysis of fluorescence quenching

Of the two tryptophan residues found in SCOMT (Trp143 and Trp38), only Trp143 is exposed and accounts for the fluorescence of SCOMT that is available for quenching. This amino acid interacts closely with the adenosine ring of SAM and keeps the planar catechol ring in the correct orientation during catalysis [46,47]. The excitation wavelength at 295 nm therefore, selectively excites the Trp143 [48], thus, this residue emitted the observed fluorescence. The binding of AgNPs caused a quenching in the tryptophanyl fluorescence of the Trp143 with linearity in the Stern–Volmer plots indicating tryptophan residue is close to the interacting site of AgNPs with SCOMT. The possible quenching mechanism can be interpreted from the analysis of the fluorescence data. This data was analyzed according to the Stern–Volmer equations (Equations (2), (3))). The Stern–Volmer constants (*K*_*SV*_) were estimated from the slopes of the linear regressions and *θ*, the fraction of tryptophan residues accessible to AgNPs, from the reciprocal of the y-intercept (Fig. 4B).

(2) Fo/F = 1 + KsvQ

(3) Fo/(Fo−F)=1/(θKsvQ)+1/θ

The ‘y-intercepts’ of the Stern–Volmer plots (Fig. 4B) were equivalent to 1, signifying the fluorescence quenching was only caused by the internal quenching of the NP and not the interplay of other fluors in solution that would result in external quenching. The *K*_*SV*_ values decreased with increasing temperature (Table 2), illustrating that the fluorescent quenching of SCOMT by AgNPs is governed by the static mechanism, supporting the formation of a non-fluorescent NP–fluorophore complex [45,49]. Similar observations were made on the Stern–Volmer analysis on the interaction of AgNPs with protein (arginine kinase) by our research group [45].

**Table 2:** Values for the fluorescent parameters (K); *K*_*SV,*_*θ, K*_*a*_, *K*_d*,*_*n* and thermodynamic parameters *ΔH*, *ΔS* and *ΔG* for the interaction of AgNPs with SCOMT.

Temperature *(K)*	*K*_*SV*_*(μM)*	Θ	*K*_*a*_*(μM*^*-1*^*)*	*K*_d_*(μM)*	*n*	*ΔG (kJ.mol*^*-1*^*.K*^*-1*^*)*
*298*	*6.2 × 10*^*-2*^	*0.561*	*4.35 × 10*^*-1*^	*2.30*	*0.51*	*−82.25*
*303*	*4.5 × 10*^*-2*^	*0.583*	*1.58 × 10*^*-1*^	*6.32*	*0.67*	*−83.00*
*308*	*3.3 × 10*^*-2*^	*0.595*	*1.15 × 10*^*-1*^	*8.70*	*0.91*	*−83.75*
*313*	*3.0 × 10*^*-2*^	*0.601*	*2.33 × 10*^*-2*^	*42.83*	*1.41*	*−84.50*

*ΔH = −3.76 × 10^4^(J.mol^-1^.K^-1^).

**ΔS = 1.50 × 10^2^(J.mol^-1^.K^-1^).

##### 3.4.2.3 Thermodynamic analysis

To elucidate the interacting forces between AgNPs and SCOMT, the temperature-dependent thermodynamic parameters were estimated according to Gibbs thermodynamic equation (Equation (4)) and the Van’t Hoff equation (Equation (5)) [50].

(4) ΔG=ΔH−TΔS

(5) $$In\left(Ksv_{2}/Ksv_{1}\right)=\Delta H/R\left[1/T_{1}-1/T_{2}\right]$$

where *R* is the gas constant (8.314 J mol^−1^ K^−1^); Δ*H* is the change in enthalpy, Δ*S* is the change in entropy, ΔG is the Gibbs (free) energy and *K*_*SV*_ is the Stern–Volmer binding constant at the corresponding temperatures.

The change in enthalpy (Δ*H*) was estimated from the slope of the linear regression (Fig. 4C) and the change in entropy (Δ*S*) was calculated from the product of y-intercept and gas constant (*R*) [51]. The mode of interaction between any ligand and protein can be determined according to the magnitudes of Δ*H* and Δ*S*. Positive values for both Δ*H* and ΔS are indicative of hydrophobic forces; their negative values indicate Van der Waals interactions and hydrogen bonds, while a negative value for Δ*H* and a positive value for Δ*S* reflect electrostatic interactions [52]. Accordingly, the negative Δ*H* and positive Δ*S* values from the interaction of AgNPs with SCOMT imply that the binding was driven by electrostatic forces and the negative Δ*G* value signified that the interaction was spontaneous and governed by large thermodynamically favorable entropies, confirmed by the large positive value of Δ*S* (Table 2).

##### 3.4.2.4 FTIR spectroscopic analysis

**Fig. 5:**
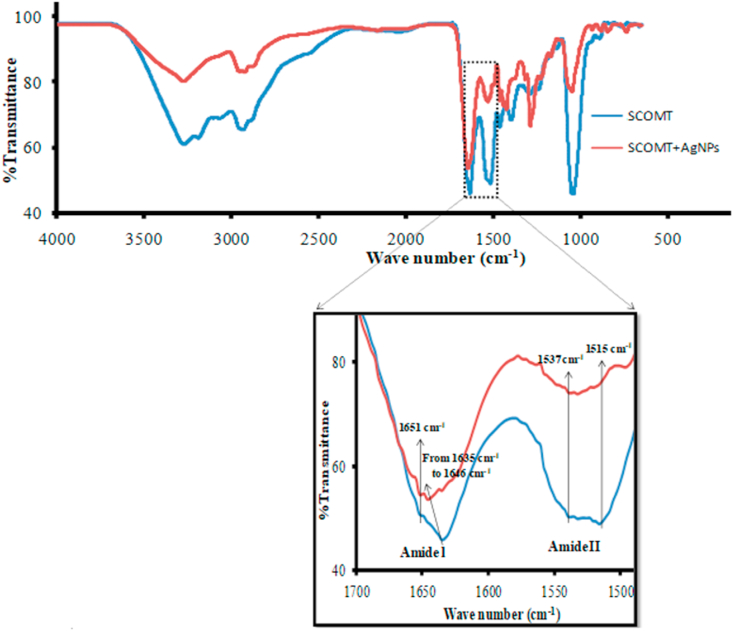
FTIR spectra of the lyophilized human SCOMT (0.5 mg) in the absence and presence of [60 μM] AgNP. Inset is the enlarged view of the amide I and II bands, showing a shift of amide I region at 1 635 cm^-1^ (to 1 646 cm^-1^), indicating relaxation of β-sheet structures.

The characteristic structure of the SAM-dependent human SCOMT comprises a seven-stranded β-sheet core (3↑2↑1↑4↑5↑7↓6↑) sandwiched between two sets of three α-helices [53]. To investigate whether the interaction with the AgNPs changes the conformation of SCOMT, FTIR measurements were performed in the absence and presence of the NPs. The FTIR spectrum of SCOMT exhibited a strong peak for amide I band (1 600–1 700 cm^-1^), which originates from an overlapping component of bands from α-helices, β-sheets, β-turns and random coil structures, hence it is sensitive to changes of protein conformation (Fig. 5) [54]. Peaks with maxima at ~1 650 cm^-1^ arise from α-helices and ~ 1 630 to ~1 635 cm-^1^ arise from β-sheet structures [55]. The adsorption of the pure SCOMT onto the NPs caused a vibrational shift of the amide I band at the β-sheets’ peak position from 1 635 to 1 646 cm^-1^ (Fig. 5), indicating that the AgNPs adsorption onto SCOMT results in the relaxation of the β-sheet structures within the enzyme. The overall decrease in transmittance in the AgNP-SCOMT complex can be attributed to the overall relaxation of the protein conformational structure caused by its adsorption onto the NPs surfaces as a function of changes in out-of-plane motions in the enzyme’s structure [55]. However, there is no noticeable shift in the peak at 1 651 cm^-1^, which originates from α-helices, suggesting that the adsorption does not cause relaxation of the α-helix structures within the enzyme.

### 3.5 Theoretical simulation: molecular docking study

**Fig. 6:**
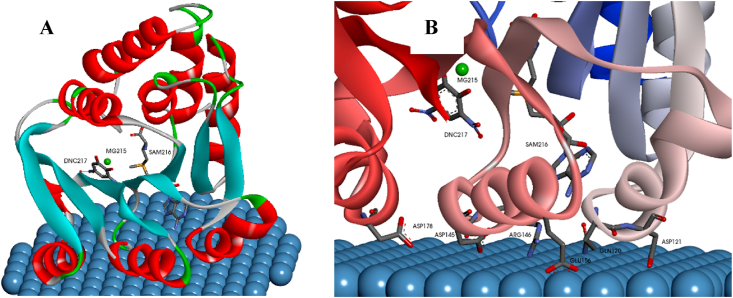
(A) Docked geometry of human SCOMT onto AgNPs showing (A) the “crawling” like structure of SCOMT onto the AgNPs surface and (B) amino acid residues that interact with the surface of the AgNPs. Positions of Mg, S-adenosyl-*L*-methionine and 3,5-dinitrocatechol ligand are shown in both images.

The computational geometrical fitting and orientation of protein onto the NPs surfaces depend on the particle curvatures (sizes), and the protein molecular structure and size [35]. It is of interest that the preferential interaction of SCOMT was reproducibly with the planar (all of the best docking poses), and not the curved portion (radius 30.0 Å) of the sphere cap. The SCOMT recognizes the Ag NPs as a flat surface and appear to “crawl” on the NP surface (Fig. 6A). The “crawling-like structures” of different proteins docked on the surfaces of NPs have been reported previously [35,56,57].

The footprint of SCOMT on the surface of AgNPs shows that the binding was facilitated by six amino acid residues, from four of the predominant helical structures (Fig. 6B), where four binding regions are involved in this interaction. The first region consists of the binding of Gln120 and Asp121 (α-helix 7); the second region involves the binding of Asp145 and Arg146 (3_10_ helix, helix 10); the third region is the binding of Glu156 (α-helix 11) and the fourth region is the binding of Asp178 (α-helix 13). All the binding distances between the participating amino acid residues and the NP surface were less than 3.5 Å (Table 3). The short distance (≤6.0Å) suggests a very strong association between the NP and the amino acid residues [58]. The estimated *ΔG* and inhibition constant (*K*_*i*_) values from the molecular docking study were −3.74 kcal mol^-1^ and 1.82 mM, respectively.

**Table 3:** The binding distances of AgNP–SCOMT participating amino acid residues.

Amino acid residue	Interaction distance (Å)
Gln120	2.72
Asp121	2.50
Asp145	2.29
Arg146	2.66
Glu156	2.82
Asp178	3.19

Docking of esculetin showed that it binds in the same position as the inhibitor 3,5-dinitrocatechol in the active site, with close proximity and coordination of one oxygen to the magnesium ion (Supplementary Fig. S5). Although the binding of the AgNP's is close to this site, because of the size of the AgNPs, it is not possible for them to dock within the protein.

The functional groups from capping, stabilizing, and/or dispersing agents on the surfaces of the NPs affect the particles’ interaction with biomolecules and, in particular, play an important role in the formation of NPs-protein interaction [59]. The variations between experimentally determined and computer modelled interactions are, most likely, due to the presence of a stabiliser (PVP) on the surfaces of AgNPs with respect to the thermodynamic results (from the fluorescence analyses). This stabiliser was omitted during the computer simulations.

## 4 Conclusion

An investigation of the interaction of SCOMT with AgNPs was conducted using spectroscopic and simulation analysis. The observed binding mechanism was a spontaneous static quenching, driven by a thermodynamically favorable positive Δ*S*, and negative Δ*H* and Δ*G* values that indicated electrostatic forces in operation. Fluorescence and Stern-Volmer analysis indicated binding of AgNPs with Trp143 and simulation analysis showed strong interaction between the NP with the enzyme protein, and leads to a relaxation of the β-sheets within the enzyme structure. The inhibition of the enzyme is dependent on concentration of the AgNPs as well as their size (<10 nm). *In silico* findings suggest the interaction of 6 amino acids from the enzyme’s helical structures. Although the exact mechanism of inhibition is unclear from this study, these findings are indicative of the potentials of AgNPs for the regulation of SCOMT activity and thus potential management of Parkinson’s disease.

## Author contributions

BSW and CGW assisted with experimental design of the research and supervised AU through his PhD studies. KL assisted with all computational experiments and BIP provided expertise and guidance on the enzymology component of the research. AU performed the laboratory experiments and writing of the main body of the manuscript. All authors were involved in advising on the research and editing of the manuscript.

## Declaration of competing interest

We declare that there are no conflicts of interest in the research submitted to the journal Biochemistry and Biophysics Reports in the manuscript entitled “Interaction of silver nanoparticles with catechol O-methyltransferase: spectroscopic and simulation analyses”
