# Supplementary material for: Interaction of silver nanoparticles with catechol O-methyltransferase: Spectroscopic and simulation analyses
Source: Biochem Biophys Rep. 2021 May 11;26:101013. doi: 10.1016/j.bbrep.2021.101013 (PMC8131974; doi:10.1016/j.bbrep.2021.101013)
Supplement: Multimedia component 1 [file mmc1.docx]

**Supplementary Data:**


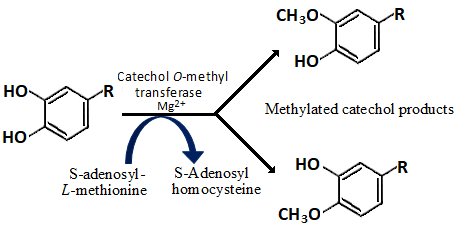


**Figure S1:** Enzymatic reaction for COMT (adapted from [21]).

10 20 30 40 50 60 70 80 90 100

....|....|....|....|....|....|....|....|....|....|....|....|....|....|....|....|....|....|....|....|

**REFERENCECATATGGGCGATACCAAAGAACAGCGTATTCTGAATCATGTTCTGCAGCATGCCGAACCGGGTAATGCACAGAGCGTTCTGGAAGCAATTGATACCTATT**

**SCOMT1 CATATGGGCGATACCAAAGAACAGCGTATTCTGAATCATGTTCTGCAGCATGCCGAACCGGGTAATGCACAGAGCGTTCTGGAAGCAATTGATACCTATT**

**SCOMT2 CATATGGGCGATACCAAAGAACAGCGTATTCTGAATCATGTTCTGCAGCATGCCGAACCGGGTAATGCACAGAGCGTTCTGGAAGCAATTGATACCTATT**

**SCOMT3 CATATGGGCGATACCAAAGAACAGCGTATTCTGAATCATGTTCTGCAGCATGCCGAACCGGGTAATGCACAGAGCGTTCTGGAAGCAATTGATACCTATT**

**SCOMT4 CATATGGGCGATACCAAAGAACAGCGTATTCTGAATCATGTTCTGCAGCATGCCGAACCGGGTAATGCACAGAGCGTTCTGGAAGCAATTGATACCTATT**

110 120 130 140 150 160 170 180 190 200

....|....|....|....|....|....|....|....|....|....|....|....|....|....|....|....|....|....|....|....|

**REFERENCEGTGAACAGAAAGAATGGGCCATGAATGTGGGTGATAAGAAAGGCAAAATTGTGGATGCCGTGATCCAAGAACATCAGCCGAGCGTGCTGCTGGAACTGGG**

**SCOMT1 GTGAACAGAAAGAATGGGCCATGAATGTGGGTGATAAGAAAGGCAAAATTGTGGATGCCGTGATCCAAGAACATCAGCCGAGCGTGCTGCTGGAACTGGG**

**SCOMT2 GTGAACAGAAAGAATGGGCCATGAATGTGGGTGATAAGAAAGGCAAAATTGTGGATGCCGTGATCCAAGAACATCAGCCGAGCGTGCTGCTGGAACTGGG**

**SCOMT3 GTGAACAGAAAGAATGGGCCATGAATGTGGGTGATAAGAAAGGCAAAATTGTGGATGCCGTGATCCAAGAACATCAGCCGAGCGTGCTGCTGGAACTGGG**

**SCOMT4 GTGAACAGAAAGAATGGGCCATGAATGTGGGTGATAAGAAAGGCAAAATTGTGGATGCCGTGATCCAAGAACATCAGCCGAGCGTGCTGCTGGAACTGGG**

210 220 230 240 250 260 270 280 290 300

....|....|....|....|....|....|....|....|....|....|....|....|....|....|....|....|....|....|....|....|

**REFERENCETGCATATTGTGGTTATAGCGCAGTTCGTATGGCACGTCTGCTGAGTCCGGGTGCACGTCTGATTACCATTGAAATTAACCCGGATTGTGCAGCAATTACC**

**SCOMT1 TGCATATTGTGGTTATAGCGCAGTTCGTATGGCACGTCTGCTGAGTCCGGGTGCACGTCTGATTACCATTGAAATTAACCCGGATTGTGCAGCAATTACC**

**SCOMT2 TGCATATTGTGGTTATAGCGCAGTTCGTATGGCACGTCTGCTGAGTCCGGGTGCACGTCTGATTACCATTGAAATTAACCCGGATTGTGCAGCAATTACC**

**SCOMT3 TGCATATTGTGGTTATAGCGCAGTTCGTATGGCACGTCTGCTGAGTCCGGGTGCACGTCTGATTACCATTGAAATTAACCCGGATTGTGCAGCAATTACC**

**SCOMT4 TGCATATTGTGGTTATAGCGCAGTTCGTATGGCACGTCTGCTGAGTCCGGGTGCACGTCTGATTACCATTGAAATTAACCCGGATTGTGCAGCAATTACC**

310 320 330 340 350 360 370 380 390 400

....|....|....|....|....|....|....|....|....|....|....|....|....|....|....|....|....|....|....|....|

**REFERENCECAGCGTATGGTTGATTTTGCCGGTGTTAAAGATAAAGTTACCCTGGTTGTTGGTGCAAGCCAGGATATTATTCCGCAGCTGAAGAAAAAGTATGACGTGG**

**SCOMT1 CAGCGTATGGTTGATTTTGCCGGTGTTAAAGATAAAGTTACCCTGGTTGTTGGTGCAAGCCAGGATATTATTCCGCAGCTGAAGAAAAAGTATGACGTGG**

**SCOMT2 CAGCGTATGGTTGATTTTGCCGGTGTTAAAGATAAAGTTACCCTGGTTGTTGGTGCAAGCCAGGATATTATTCCGCAGCTGAAGAAAAAGTATGACGTGG**

**SCOMT3 CAGCGTATGGTTGATTTTGCCGGTGTTAAAGATAAAGTTACCCTGGTTGTTGGTGCAAGCCAGGATATTATTCCGCAGCTGAAGAAAAAGTATGACGTGG**

**SCOMT4 CAGCGTATGGTTGATTTTGCCGGTGTTAAAGATAAAGTTACCCTGGTTGTTGGTGCAAGCCAGGATATTATTCCGCAGCTGAAGAAAAAGTATGACGTGG**

410 420 430 440 450 460 470 480 490 500

....|....|....|....|....|....|....|....|....|....|....|....|....|....|....|....|....|....|....|....|

**REFERENCEATACCCTGGATATGGTGTTTCTGGATCATTGGAAAGATCGTTATCTGCCGGATACCCTGCTGCTGGAAGAATGTGGTCTGCTGCGTAAAGGCACCGTTCT**

**SCOMT1 ATACCCTGGATATGGTGTTTCTGGATCATTGGAAAGATCGTTATCTGCCGGATACCCTGCTGCTGGAAGAATGTGGTCTGCTGCGTAAAGGCACCGTTCT**

**SCOMT2 ATACCCTGGATATGGTGTTTCTGGATCATTGGAAAGATCGTTATCTGCCGGATACCCTGCTGCTGGAAGAATGTGGTCTGCTGCGTAAAGGCACCGTTCT**

**SCOMT3 ATACCCTGGATATGGTGTTTCTGGATCATTGGAAAGATCGTTATCTGCCGGATACCCTGCTGCTGGAAGAATGTGGTCTGCTGCGTAAAGGCACCGTTCT**

**SCOMT4 ATACCCTGGATATGGTGTTTCTGGATCATTGGAAAGATCGTTATCTGCCGGATACCCTGCTGCTGGAAGAATGTGGTCTGCTGCGTAAAGGCACCGTTCT**

510 520 530 540 550 560 570 580 590 600

....|....|....|....|....|....|....|....|....|....|....|....|....|....|....|....|....|....|....|....|

**REFERENCEGCTGGCAGATAATGTTATTTGTCCTGGTGCACCGGATTTTCTGGCACATGTTCGTGGTAGCAGCTGTTTTGAATGTACCCATTATCAGTCCTTTCTGGAA**

**SCOMT1 GCTGGCAGATAATGTTATTTGTCCTGGTGCACCGGATTTTCTGGCACATGTTCGTGGTAGCAGCTGTTTTGAATGTACCCATTATCAGTCCTTTCTGGAA**

**SCOMT2 GCTGGCAGATAATGTTATTTGTCCTGGTGCACCGGATTTTCTGGCACATGTTCGTGGTAGCAGCTGTTTTGAATGTACCCATTATCAGTCCTTTCTGGAA**

**SCOMT3 GCTGGCAGATAATGTTATTTGTCCTGGTGCACCGGATTTTCTGGCACATGTTCGTGGTAGCAGCTGTTTTGAATGTACCCATTATCAGTCCTTTCTGGAA**

**SCOMT4 GCTGGCAGATAATGTTATTTGTCCTGGTGCACCGGATTTTCTGGCACATGTTCGTGGTAGCAGCTGTTTTGAATGTACCCATTATCAGTCCTTTCTGGAA**

610 620 630 640 650 660 670

....|....|....|....|....|....|....|....|....|....|....|....|....|....|..

**REFERENCETATCGTGAAGTTGTTGATGGTCTGGAAAAAGCCATCTATAAAGGTCCGGGTAGCGAAGCAGGTCCGCTCGAG**

**SCOMT1 TATCGTGAAGTTGTTGATGGTCTGGAAAAAGCCATCTATAAAGGTCCGGGTAGCGAAGCAGGTCCGCTCGAG**

**SCOMT2 TATCGTGAAGTTGTTGATGGTCTGGAAAAAGCCATCTATAAAGGTCCGGGTAGCGAAGCAGGTCCGCTCGAG**

**SCOMT3 TATCGTGAAGTTGTTGATGGTCTGGAAAAAGCCATCTATAAAGGTCCGGGTAGCGAAGCAGGTCCGCTCGAG**

**SCOMT4 TATCGTGAAGTTGTTGATGGTCTGGAAAAAGCCATCTATAAAGGTCCGGGTAGCGAAGCAGGTCCGCTCGAG**

**Figure S2:** Alignments of reference SCOMT (optimized for *E. coli* expression) with four sequenced samples.


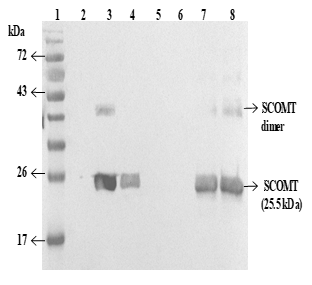


**Figure S3:** A western blot gels of samples collected after each purification stage showing the protein ladder (1), grown *E. coli* cells harbouring empty (no HSCOMT gene) plasmid (2), crude lysate sample (3), cleared lysate sample (4), IMAC flow-through sample (5), IMAC Wash sample (6), IMAC eluate sample (7) and size exclusion elute sample (8).

**Figure S4:** (A) The plasmonic absorption spectrum of AgNPs samples recorded at 2 h and 14 days after synthesis. The discontinuous line is the absorption of ethanolic PVP solution; (B) TEM image of AgNPs stored in the dark at 23^o^C, after synthesis for 2 h and (C) 14 days. The corresponding insets are the size distribution graphs shown adjacent to each TEM image. Scale bars represent 100 nm.


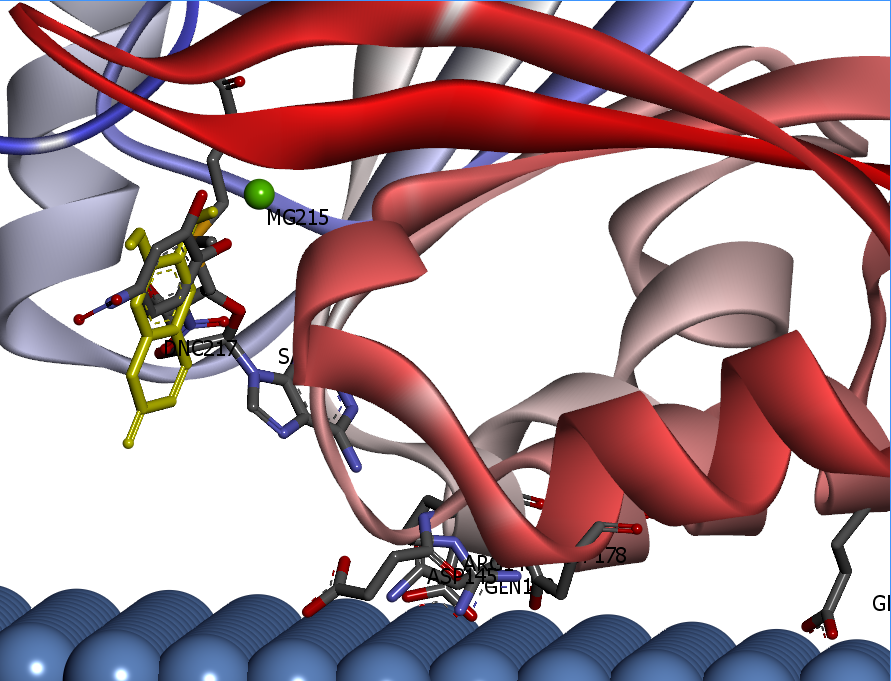


**Figure S5:** Geometry of human SCOMT onto AgNPs showing the docked position of esculetin. Esculetin docked in the same position as inhibitor, 3,5-dinitrocatechol, in the active site with close proximity and coordination (of one oxygen) to the magnesium ion. The image shows an overlay of the original ligand DNC (colored by element) with esculetin (yellow).
